# Supplementary material for: Targeted Lignan Profiling and Anti-Inflammatory Properties of Schisandra rubriflora and Schisandra chinensis Extracts
Source: Molecules. 2018 Nov 27;23(12):3103. doi: 10.3390/molecules23123103 (PMC6321394; doi:10.3390/molecules23123103)
Supplement: Supplementary file 1 [file molecules-23-03103-s001.pdf]

SUPPLEMENTARY MATERIALS

Targeted lignan profiling and anti-inflammatory properties of *Schisandra rubriflora* and *Schisandra chinensis* extracts

Agnieszka Szopa[1]*, Dziurka Michał[2], Angelika Warzecha[1], Kubica Paweł[1], Marta Klimek-Szczykutowicz[1], Ekiert Halina[1]

[1] Chair and Department of Pharmaceutical Botany, Jagiellonian University, Medical College, ul. Medyczna 9, 30-688 Kraków, Poland

[2] Polish Academy of Sciences The Franciszek Górski Institute of Plant Physiology, ul. Niezapominajek 21, 30-239 Kraków, Poland

*Corresponding author: PhD Agnieszka Szopa, phone +48 12 620 54 30, fax +48 620 54 40, e-mail: a.szopa@uj.edu.

Table S1. The standard lignan substances used in the performer studies.

No*	Lignan name	Synonymous names	Chemical classification of lignans	Molecular formula	Structural formula	Molecular weight [g/mol]
L01	6-O-Benzoylgomisin O	Benzoylgomisin O	Dibenzocyclooctadiene lignans	$C_{30}H_{32}O_8$	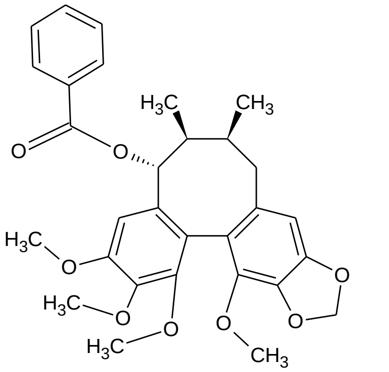	520.578
L02	Schisandrin C	Wuweizisu C, Schizandrin C	Dibenzocyclooctadiene lignans	$C_{22}H_{24}O_6$	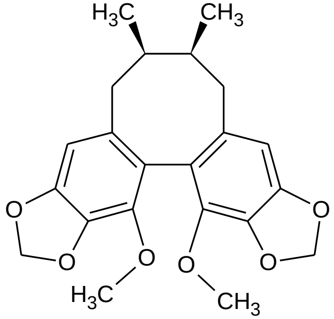	384.428
L04	Schisanhenol	Gomisin K_3	Dibenzocyclooctadiene lignans	$C_{23}H_{30}O_6$	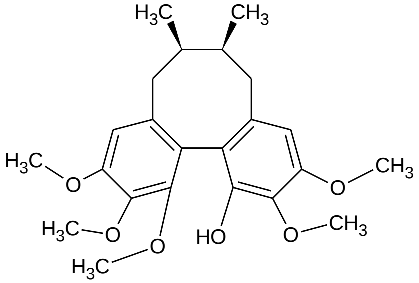	402.487

No*	Lignan name	Synonymous names	Chemical classification of lignans	Molecular formula	Structural formula	Molecular weight [g/mol]
L05	Schisantherin B	Gomisin B, Schisandrer B	Dibenzocyclooctadiene lignans	$C_{28}H_{34}O_9$	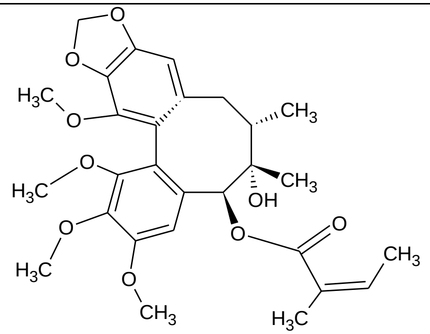	514.571
L06	Schisantherin A	Gomisin C, Schizandrer A	Dibenzocyclooctadiene lignans	$C_{30}H_{32}O_9$	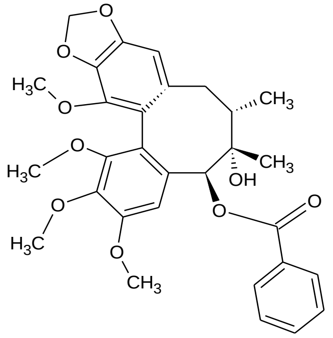	536.577
L07	Schisandrin A	Schizandrin A, Deoxyschisandrin, Deoxyschizandrin, Dimethylgomisin J	Dibenzocyclooctadiene lignans	$C_{24}H_{32}O_6$	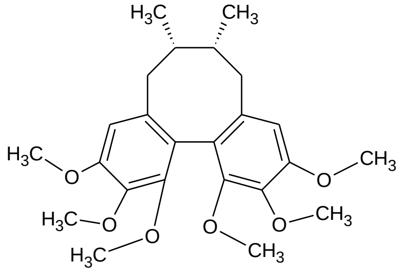	416.514

No*	Lignan name	Synonymous names	Chemical classification of lignans	Molecular formula	Structural formula	Molecular weight [g/mol]
L08	Rubrisandrin A	Arisanschinin G	Dibenzocyclooctadiene lignans	$C_{22}H_{28}O_6$	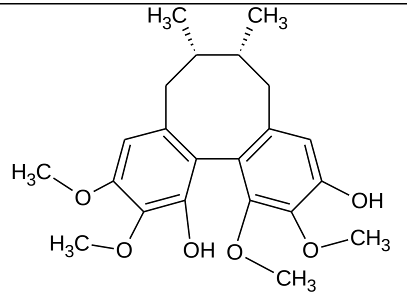	388.460
L09	Rubriflorin A	Interiotherin C	Dibenzocyclooctadiene lignans	$C_{30}H_{36}O_{10}$	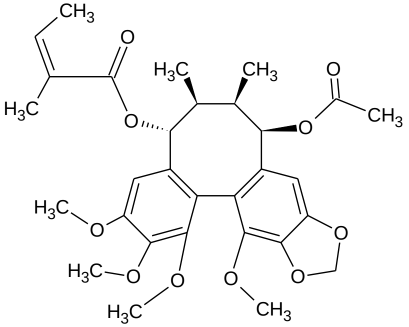	556.608
L10	Schisandrin	Schisandrine, Schizandrin, Schizandrol A, Wuweizichun A, Wuweizi alcohol A, Wuweizisu A	Dibenzocyclooctadiene lignans	$C_{24}H_{32}O_7$	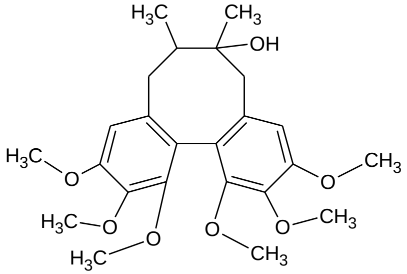	432.513

No*	Lignan name	Synonymous names	Chemical classification of lignans	Molecular formula	Structural formula	Molecular weight [g/mol]
L12	Wulignan A_1	Arisantetralone A	Aryltetralin lignans	$C_{20}H_{22}O_5$	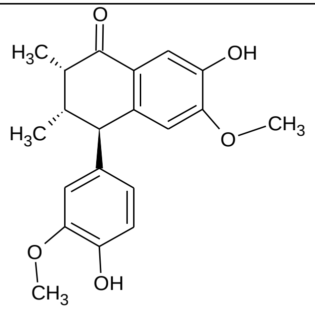	342.391
L13	Gomisin O	n.f.	Dibenzocyclooctadiene lignans	$C_{23}H_{28}O_7$	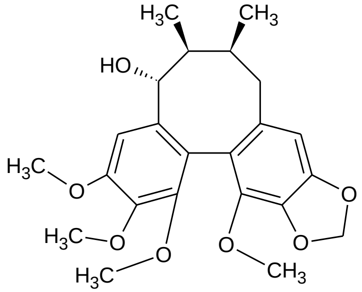	416.470
L14	Gomisin N	Isokadsuranin, Deoxygomisin A	Dibenzocyclooctadiene lignans	$C_{23}H_{28}O_6$	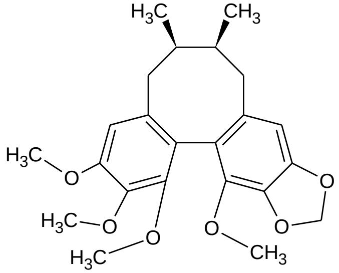	400.471

No*	Lignan name	Synonymous names	Chemical classification of lignans	Molecular formula	Structural formula	Molecular weight [g/mol]
L15	Gomisin J	n.f.	Dibenzocyclooctadiene lignans	$C_{22}H_{28}O_6$	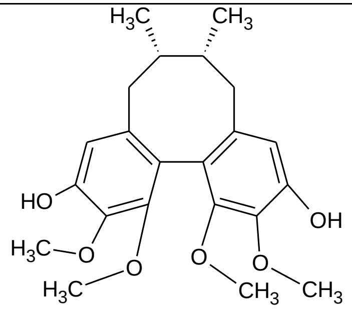	388.460
L16	Gomisin D	n.f.	Dibenzocyclooctadiene lignans	$C_{28}H_{34}O_{10}$	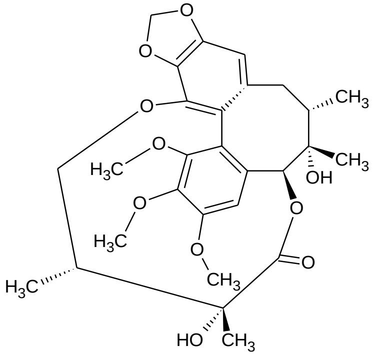	530.570
L17	Gomisin A	Schizandrol B, Besigomisin, Schisandrol B, Wuweizi alcohol B	Dibenzocyclooctadiene lignans	$C_{23}H_{28}O_7$	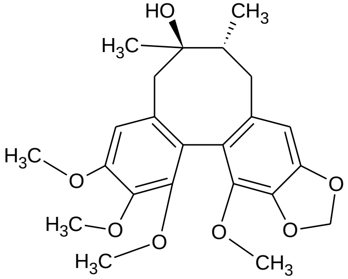	416.470

No*	Lignan name	Synonymous names	Chemical classification of lignans	Molecular formula	Structural formula	Molecular weight [g/mol]
L18	Gomisin G	n.f.	Dibenzocyclooctadiene lignans	$C_{30}H_{32}O_9$	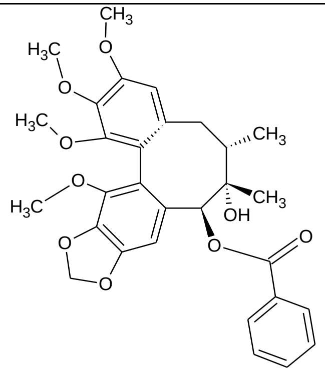	536.577
L19	Pregomisin	n.f.	Dibenzylbutane lignans	$C_{22}H_{30}O_6$	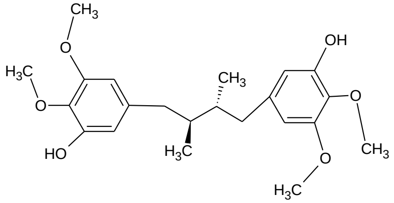	390,476
L20	Epigomisin O	Gomisin O, 6-Epigomisin O	Dibenzocyclooctadiene lignans	$C_{23}H_{28}O_7$	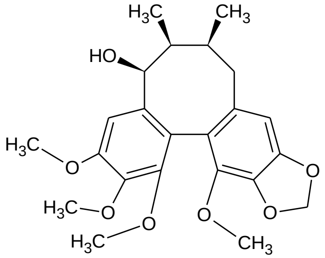	416,470
L21	Licarin A	Diisoeugenol, Dehydroisoeugenol	Dihydrobenzofuran neolignans	$C_{20}H_{22}O_4$	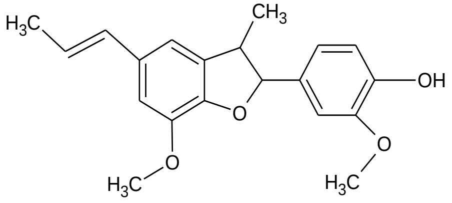	326,392

No*	Lignan name	Synonymous names	Chemical classification of lignans	Molecular formula	Structural formula	Molecular weight [g/mol]
L22	Mesodihydroguaiaretic acid	Dihydroguaiaretic acid	Dibenzocyclooctadiene lignans	$C_{20}H_{26}O_4$	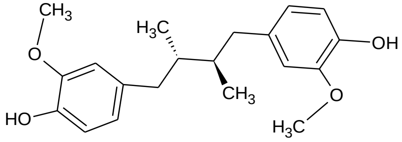	330,424
L23	Fragransin A_2	Nectandrin B	Tetrahydrofuran lignans	$C_{20}H_{24}O_5$	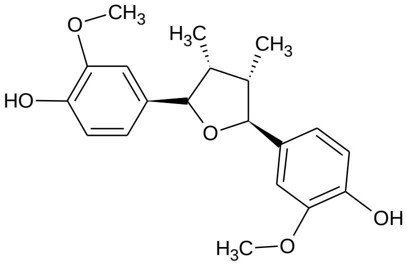	344,407
L26	Licarin B	Licarine B	Dihydrobenzofuran neolignans	$C_{20}H_{20}O_4$	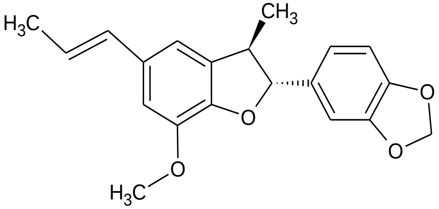	324,376

Structural formulas drown in: ACD/ChemSkech (Freeware), version 12.00, Advanced Chemistry Development, Inc., Toronto, ON, Canada, www.acdlabs.com, 2010.

* No – number of compound used for elaboration of results; corresponds with Figure S1 and Table S2

n.f. – has not been found

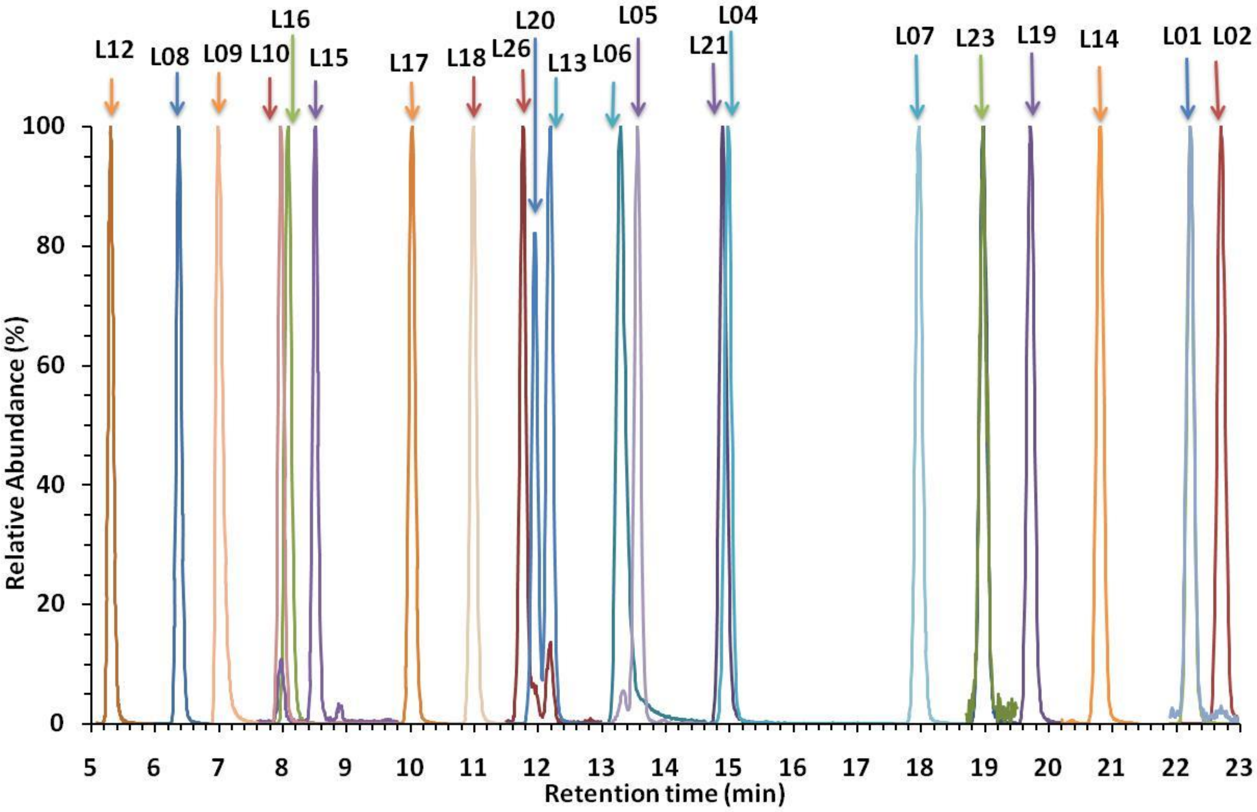

Figure S1. Exemplary UHPLC – MS/MS MRM chromatogram of lignan standard mixture at 100 ug/ml (corresponding with Table S1 and S2)

Table S2. The monitored fragmentation reactions (multiple reactions monitoring, MRM) for studied lignans. Ionization conditions applied: positive ionization (+ESI), drying gas temperature - 350°C, gas flow - 12 l/min, nebulizer pressure - 35 psi (Corresponding with Table S1 and Figure S1)

Lignans	No*	Monoisotopic Mass (Da)	Type of ion	Quantifier Transition	Fragmentor Voltage (V)	Collision energy (V)	Retention time (min)	Polarization	MRM Time Segment
Wulignan A_1	L12	342.1	[M+H]+	343.3\117.0	99	90	5.31	Positive	2
Rubrisandrin A	L08	388.2	[M-19]+	369.3\351.1	109	5	6.37	Positive	3
Rubriflorin A	L09	556.2	[M+H]+	557.4\173.0	185	65	6.99	Positive	4
Schisandrin	L10	432.2	[M-H2O+H]+	415.3\359.2	150	13	7.97	Positive	5
Gomisin D	L16	530.2	[M-C6H10O3+H]+	401.3\168.0	150	77	8.52	Positive	5
Gomisin J	L15	388.2	[M+H]+	389.3\117.0	150	77	8.90	Positive	5
Pregomisin	L19	390.2	[M+H]+	391.3\139.0	150	61	19.71	Positive	5
Gomisin N	L14	400.2	[M+H]+	401.3\168.0	150	77	20.81	Positive	5
Gomisin A	L17	416.2	[M-H2O+H]+	399.3\368.2	100	150	10.03	Positive	6
Epigomisin O	L20	416.2	[M-H2O+H]+	399.3\368.2	100	150	11.95	Positive	6
6-O-Benzoylgomisin O	L01	520.2	[M-C7H6O2+H]+	399.3\368.2	100	150	22.22	Positive	6
Gomisin G	L18	536.2	[M-C7H6O2+H]+	415.3\371.1	150	13	10.99	Positive	7
Schisantherin A	L06	536.2	[M-C7H6O2+H]+	415.3\371.1	150	13	13.56	Positive	7
Schisantherin B	L05	514.2	[M-C5H8O2+H]+	415.3\371.1	150	13	13.97	Positive	7
Licarin B	L26	324.1	[M+H]+	325.2\152.0	100	130	11.75	Positive	8
Gomisin O	L13	416.2	[M-H2O+H]+	399.3\368.2	100	130	12.20	Positive	8
Mesodihydroguaiaretic acid	L22	330.2	[M+H]+	331.3\117.0	130	53	13.29	Positive	9
Dehydroisoeugenol	L21	326.2	[M+H]+	327.3\105.1	150	130	14.90	Positive	10
Schisanhenol	L04	402.2	[M+H]+	403.3\231.0	150	130	14.98	Positive	10
Schisandrin A	L07	416.2	[M+H]+	417.3\316.2	160	21	17.97	Positive	11
Fragransin A_2	L23	344.2	[M-21]+	323.2\152.0	100	81	18.98	Positive	12
Schisandrin C	L02	384.2	[M+H]+	385.3\231.1	130	17	22.70	Positive	16

*No -number of compound used for elaboration of results; corresponds with Figure S1 and Table S1

Table S3. Quantitative composition of "average sample lignan composition" (MIX) at 1.75 μg/ml

**Compound No***	S5	S1	S10	S16	S14	S7	S6	S4	S18	S15	S13	S2	S20	S22	S12	S21
**Mol Fraction (%)**	14.30	13.70	11.10	8.00	11.60	8.10	11.80	5.40	3.90	4.40	2.70	2.90	0.60	0.90	0.30	0.30

No* - corresponding with Tabl
